# A TMA De-Arraying Method for High Throughput Biomarker Discovery in Tissue Research

**DOI:** 10.1371/journal.pone.0026007

**Published:** 2011-10-07

**Authors:** Yinhai Wang, Kienan Savage, Claire Grills, Andrena McCavigan, Jacqueline A. James, Dean A. Fennell, Peter W. Hamilton

**Affiliations:** Centre for Cancer Research and Cell Biology, Queen's University Belfast, Belfast, United Kingdom; Dana-Farber Cancer Institute, United States of America

## Abstract

**Background:**

Tissue MicroArrays (TMAs) represent a potential high-throughput platform for the analysis and discovery of tissue biomarkers. As TMA slides are produced manually and subject to processing and sectioning artefacts, the layout of TMA cores on the final slide and subsequent digital scan (TMA digital slide) is often disturbed making it difficult to associate cores with their original position in the planned TMA map. Additionally, the individual cores can be greatly altered and contain numerous irregularities such as missing cores, grid rotation and stretching. These factors demand the development of a robust method for de-arraying TMAs which identifies each TMA core, and assigns them to their appropriate coordinates on the constructed TMA slide.

**Methodology:**

This study presents a robust TMA de-arraying method consisting of three functional phases: TMA core segmentation, gridding and mapping. The segmentation of TMA cores uses a set of morphological operations to identify each TMA core. Gridding then utilises a Delaunay Triangulation based method to find the row and column indices of each TMA core. Finally, mapping correlates each TMA core from a high resolution TMA whole slide image with its name within a TMAMap.

**Conclusion:**

This study describes a genuine robust TMA de-arraying algorithm for the rapid identification of TMA cores from digital slides. The result of this de-arraying algorithm allows the easy partition of each TMA core for further processing. Based on a test group of 19 TMA slides (3129 cores), 99.84% of cores were segmented successfully, 99.81% of cores were gridded correctly and 99.96% of cores were mapped with their correct names via TMAMaps. The gridding of TMA cores were also extensively tested using a set of 113 pseudo slide (13,536 cores) with a variety of irregular grid layouts including missing cores, rotation and stretching. 100% of the cores were gridded correctly.

## Introduction

Tissue MicroArrays (TMAs) represent a potential high-throughput platform for the analysis and discovery of tissue biomarkers, diagnostic support and patient targeted therapies [1]. The technique allows hundreds of individual tissue samples to be hosted on a single glass slide, which can be labelled for a target biomarker with chromogenic or fluorescence labels and scored to determine the relationship between the presence of the biomarker and diagnosis, prognosis or response to therapy. With the emergence of commercial slide scanners, TMA slides can be scanned, in their entirety, as high resolution (0.25 µm/pixel) digital images, called virtual slides (*aka*. digital slides). This has enabled researchers to analyse each single TMA core using various computer-based, software analysis systems more rapidly and objectively [2], [3], [4]. However, a bottleneck and technical challenge for TMA image analysis is the automated recognition of single tissue cores within a TMA virtual slide that may contain hundreds of individual cores. It is important to properly assign individual cores to their appropriate array (row and column) position, as this is how the core sample is identified and associated with its relevant clinical and pathological metadata. This is generally performed manually which is extremely tedious and time consuming. For this reason, the development of an automated method to “de-array” TMAs and accurately assign array positions to cores would both save time and potentially increase TMA scoring output. Successful automated TMA de-arraying would facilitate high-throughput TMA experiments using computer based image processing and machine vision techniques by eliminating the cumbersome manual de-arraying process and enable rapid batch processing e.g. biomarker quantification with respect to individual core clinical characteristics [4].

TMA de-arraying refers to a procedure which firstly segments each TMA core from the original TMA virtual slide, finds the 2D grid index of each tissue core in the 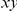-plane and maps these to the associated metadata with the cores. Core identifiers (names) and associated clinical and pathological data are generally stored in an anonymised database or a spreadsheet. Ultimately, a TMA de-arraying platform should consolidate information regarding the TMA core's 2D grid index, with TMA core names (and the associated patient data) with the actual TMA images.

TMA de-arraying is a challenging problem. The layout of TMA cores is theoretically in the form of a regular grid. Nonetheless, the reality is that TMA slides rarely represent regular 2D arrays with consistent spacing between cores. This is due to the fact that the labile nature of the TMA means that it is easily, and often, altered during slide preparation and processing. For example, the layout can be rotated or stretched, etc. Furthermore, tissue cores can also be fragmented. Some tissue cores can also be lost. These imperfections, which are inherent in TMA production and slide processing, greatly contribute to the complex, noisy 2D image data associated with digital TMAs. Though TMA de-arraying appears to be easy to the naked eye, the successful automated de-arraying of the majority of TMA cores can be challenging in image processing and computer graphics especially when rare and difficult cases, such as severely stretched grid layout arise.

Surprisingly, relatively few studies describe de-arraying methods [4], [5], [6], [7], [8], [9], [10]. For the segmentation of TMA cores, a number of groups have used simple thresholding based methods on image intensities [6], [8], [9]. One study used a multi-step approach utilising a number of image processing techniques, including the use of K-means for grouping fragmented tissue cores [10]. Importantly these studies presented their segmentation methods using very descriptive terms, and did not report the performance and/or accuracy of their methods. Therefore it is difficult to replicate their algorithms and to make objective judgements of how robust they are. Fortunately we are able to utilise a variety of image segmentation techniques, such as image morphology, watershed, active contour and statistical modelling methods which are generally employed for the segmentation of round objects from white background, e.g. the segmentation of cell nuclei [11], [12], [13], [14]. Though the segmentation of TMA cores and nuclei are similar, the unique morphological characteristics of TMA cores such as broken and/or distorted cores needs to be considered when generating a segmentation algorithm. This has been attempted by Rabinovich et. al. by the use of a K-means method to connect/disconnect tissue parts within a TMA core [10], however it did not evaluate how robust this method is. Alternatively, Dell'Anna et. al. reported an accuracy of 96.84% from 5878 cores [5]. It used a simple algorithm for the gridding of TMA cores by calculating distance between neighbouring cores. However, this heavily relies on the existence of “complete rows” within TMA sections. Without complete rows, this proposed method tends to produces severe gridding errors. Another group used a Hough transform [15] based approach for the detection of straight lines within TMA section grid patterns, however details of the algorithm are not presented and the accuracy of the proposed algorithm is not reported either. Such algorithms are also susceptible to situations where large numbers of cores are missing [6], [7]. Thallinger et al. introduced a complete TMA data management and analysis framework, however it requires initial human interaction to perform de-arraying and the details of the algorithm are not presented [16]. Furthermore, the de-arraying algorithm is not evaluated either. Another study by Lahrmann et al. describes a template matching approach for the gridding of TMA cores. However, it is unclear how the initial grid template is designed and although it reports a gridding accuracy of 99.59% using 60 slides containing 8900 cores, it does not demonstrate that the proposed simple Euclidian distance and thresholding based grid matching approach works with sparse grids (e.g. with many missing cores) or severely altered (rotated and stretched) grid layouts. Nevertheless all of these studies failed to disclose enough technical implementation details in order to regenerate their reported de-Arraying algorithms. Additionally, none of these reports have compared the performance of their algorithms with commercially available de-arraying software packages such as Aperio's Spectrum and Definiens' TissueStudio and each of the two software packages (cost >£10,000 in year 2011).

Therefore, in this study, we introduce a robust TMA de-arraying algorithm which contains three functional partitions, Segmentation, Gridding and Mapping. In addition we provide explicit technical details of the method and have evaluated the performance of our algorithm against other studies as well as currently available commercial platforms using both real TMA slide data as well as artificially generated pseudo TMA slide.

## Materials and Methods

### Ethics statement

This study was approved by the Office for Research Ethics Committees Northern Ireland (ORECNI). REC reference: 06/NIR01/94. ORECNI waived the need for patient consent as the samples were accessed retrospectively from the NHS pathology archives of patients who had been treated for lung cancer up to 10 years earlier. The image data derived from the patient TMA slide were analysed entirely anonymously.

### Materials

A collection of 19 TMA slides were generated, stained and processed within the Tissue Core Technology Unit at the Centre for Cancer Research and Cell Biology, Queens University Belfast. A large amount of these samples are used as a part of a large on-going project with our centre for the investigation of novel biomarkers from non-small cell lung cancer for drug discovery and targeted therapies. Beside the routine H&E stain, these slides have also been stained with a variety of biomarkers using Immunohistochemistry (IHC DAB), including the BCL-2 family proteins BAK [17], BAX [18] and NOXA [19], a novel putative cancer biomarker, named CB1. Negative control TMAs were also generated by staining with secondary antibody alone (i.e. the same procedure without primary antibody). Taken together these slides encompass 3246 TMA cores, however a total of 115 cores were missing and 2 cores are significantly partitioned into a number of fragments. This results in a total of 3129 valid TMA cores, representing 96.40% of all the TMA cores. Seventeen of these slides have TMA cores with a diameter of 600 µm, with the remaining two having core diameters of 1200 µm and 500 µm respectively. Details of the three virtual slides are listed in Table 1. Slides with ID from 1 to 17 are also accompanied with a TMAMap, which are Microsoft Excel files containing all TMA core names and associated anonymised clinical information.

**Table 1:** The Details of TMA Virtual Slides Used for Testing.

ID	Tissue Type	Stain or Biomarker	Core Diameter	Total Cores	Missing Cores	Fragmented Cores	Remaining Cores	%
**1**	Lung	BAK	600 µm	232	6	0	226	97.41%
**2**	Lung	BAX	600 µm	232	5	0	227	97.84%
**3**	Lung	H&E	600 µm	232	3	0	229	98.71%
**4**	Lung	CB1	600 µm	232	7	2	223	96.12%
**5**	Lung	Negative Control	600 µm	232	8	0	224	96.55%
**6**	Lung	NOXA	600 µm	232	7	0	225	96.98%
**7**	Lung	BAK	600 µm	114	0	0	114	100%
**8**	Lung	BAX	600 µm	114	0	0	114	100%
**9**	Lung	H&E	600 µm	114	0	0	114	100%
**10**	Lung	CB1	600 µm	114	0	0	114	100%
**11**	Lung	Negative Control	600 µm	114	0	0	114	100%
**12**	Lung	NOXA	600 µm	114	0	0	114	100%
**13**	Lung	BAX	600 µm	144	4	0	140	97.22%
**14**	Lung	H&E	600 µm	144	1	0	143	99.31%
**15**	Lung	CB1	600 µm	144	2	0	142	98.61%
**16**	Lung	Negative Control	600 µm	144	4	0	140	97.22%
**17**	Lung	NOXA	600 µm	144	1	0	143	99.31%
**18**	Brain	unknown	1200 µm	90	10	0	80	88.89%
**19**	unknown	unknown	500 µm	360	57	0	303	84.17%
**Total**				3,246	115	2	3,129	96.40%

These 19 slides were subsequently scanned using an Aperio ScanScope CS whole slide scanner at 40× magnification using the objective of 20×/0.75 Plan Apo with a doubler. Virtual slide images generated have a resolution of 0.25 µm/pixel. After scanning, TMA virtual slides were compressed using lossy JPEG compression at the compression quality of 70.

### TMA Core Segmentation

**Figure 1:**
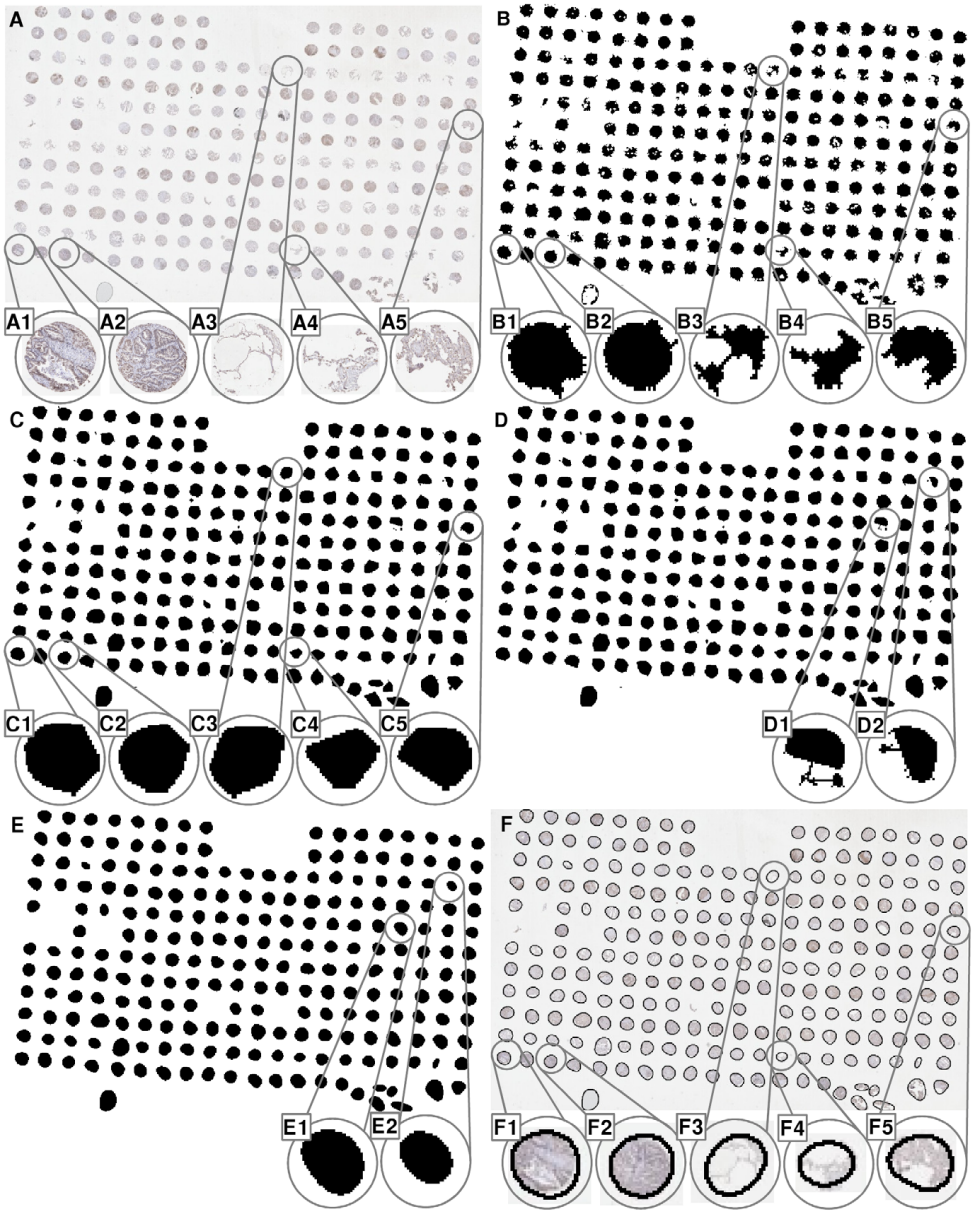
An example of TMA core segmentation procedure. (A) A down-sampled CB1 stained lung tissue TMA slide, 1072×667 pixels, on average each core has the diameter of 700 µm which gives the down-sampled core of the diameter of 30 pixels, (A1)–(A5) enlarge view of 5 cores, (B) A basic binary mask, (C) After performing convex hull operation, (D) Connecting broken TMA cores with straight lines, (E) After performed a pair of forward and inverse Fourier descriptors, (F) To superimpose the boundaries of segmented TMA core on top of the slide thumbnail. *For all the binary images in Figure B–E, colour is inversed to highlight details.

To reduce processing time, scanned TMA virtual slides were down-sampled to generate an image of approximately 1,000×1,000 pixels (i.e. 0.01% of their original size), with TMA cores being approximately 20–50 pixels in diameter. An example is shown in Figure 1A.

Firstly, the contrast of thumbnail images are enhanced using Contrast-limited adaptive histogram equalization (CLAHE) [20], and further enhanced using the negative of the Laplacian filter with 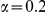, followed by Otsu's histogram based global thresholding [21] and morphological close operation. A basic TMA core binary mask is presented (Figure 1B). Each binary object (potential TMA core) is then converted to a convex hull 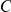 [22].

Convex hull is defined as following. Given a set of pixels 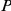 in 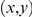 plane, if and only if, when pixel 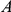 and 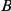 are in 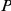, all pixels on the line segment 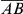 must also be presented in 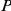. The convex hull 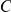 used in this study is in fact the smallest convex set, which is the intersection of all 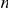 convex sets.
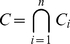
(1)It is an important step to perform convex hull transformation especially for the TMA cores which have little tissue contents, however have certain parts of the tissue core skeleton, such as the examples shown in Figure 1B. Small binary objects, which are mainly artefacts especially at slide boundaries and have an area of less than 10 pixels, are then removed.

A single TMA core may contain two or more pieces of disconnected tissue regions, examples of such cores are shown in Figure 1C and Figure 1D. In this situation, it is unavoidable to segment one broken core as many separate objects. We have developed an area-distance approach to recognise broken cores.

Based on the results from the previous step, the entire area 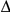 for each segmented convex object can be obtained. It is assumed that the majority of TMA cores from a single TMA slide are of a same size in terms of the core area and diameter. Therefore, by calculating the first quartile 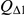 and inter-quartile range 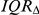 of core areas, we are able to recognise lower outliers, which are the set of TMA cores having the smallest area that satisfies:
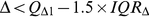
(2)By using the centroids of all potential TMA core objects as vertices and applying Delaunay Triangulation [23], we are also able to determine the distances 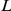 among all neighbouring cores. Typically, for a fragmented core, the distance 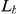 among all disconnected tissue fragments should satisfy:
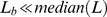
(3)For each pair of vertices 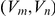 in the Delaunay Triangulation, we consider both of the vertices as the broken tissue fragments if they satisfy the follow criteria:
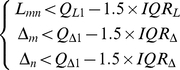
(4)where 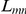 is the edge length between vertex 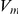 and 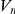, and 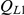 is the first quartile of all edge lengths and 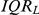 is the inter-quartile range in edge lengths.

The joining of two tissue fragments can be achieved by firstly drawing a line between the centroids of the two binary objects followed by applying the convex hull operation (examples are shown in Figure 1 D1 and D2).

The segmentation results can be further improved by removing the spur (short spike of pixels) at the boundaries of TMA cores. These spur pixels are largely artefacts introduced during contrast enhancement. The removal spur pixels uses a pair of forward and inverse Fourier descriptors [15], [24].

The boundary of a binary object (TMA core) in 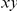-plane can be expressed with a sequence of 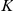 pixels: 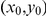, 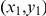,…,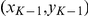 in either clockwise or anticlockwise direction. It can also be represented as a sequence of coordinates 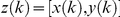 for 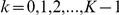. Each pair of coordinates can also be written as complex numbers so that
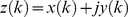
(5)By applying the Discrete Fourier Transform (DFT) on 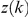, we then get the boundary Fourier descriptor
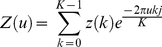
(6)for 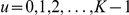. The boundary 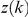 can be restored using the inverse Fourier transform:
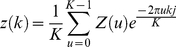
(7)In order to remove spurs, which are the high-frequency details of the boundary descriptor, we are able to use 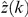, the approximation of 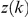:
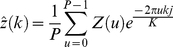
(8)for 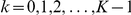, though only 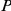 terms are used to recover 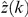. When 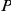 is chosen as a very small integer, the majority of high-frequency details (spurs) are removed and only low-frequency components are left to recover the global shape (near-circular shape) of TMA cores. Examples of the effect of Fourier descriptor are shown in Figure 1E.

After further removing objects smaller than 200 pixels in area, the segmentation of TMA cores is finished. Examples of overlapping the boundaries of segmentation results on the original RGB image are shown in Figure 1F.

### Gridding

#### Traveling Algorithm

Gridding is the determination of the logical coordinates of each TMA core on a virtual slide, which requires the identification of relative geometric relationships among all TMA cores. After the segmentation of TMA cores, we obtained a set of morphological features from each core, including area, centroid and bounding box in a 2D plane.

To consider all the centroids of segmented TMA cores in 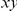-plane 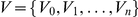, the triangulation 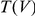 of 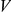 is defined as a subdivision of the 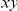-plane whose bounded faces are triangles and vertices 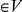. A triangulation 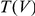 is considered as Delaunay triangulation 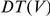 of 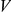 if, and only if, the circum-circle of any triangle of 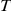 does not contain any other vertices in 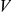 [23], [25]. Many existing methods [26], [27] can be used for the computation of Delaunay triangulation.

Delaunay triangulation is especially useful for the gridding of TMA cores. Ideally if all TMA cores are aligned in a regular 2D grid with a same core-to-core distance in both horizontal and vertical direction, and if we restrain the angles for all edges from 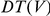 to be in the range of 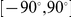, they can only form five different angles, which are:
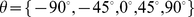
(9)Each triangle 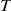 should be a right angled isosceles triangle, which contains twice as many sides than hypotenuses in 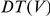. To use the rich information from Delaunay triangulation, we are able to design a travelling algorithm for the gridding of TMA core centroids. For a randomly identified start point 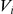, we are able to find its immediate neighbour 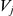 in either horizontal/vertical or clockwise/anti-clockwise direction.

**Figure 2:**
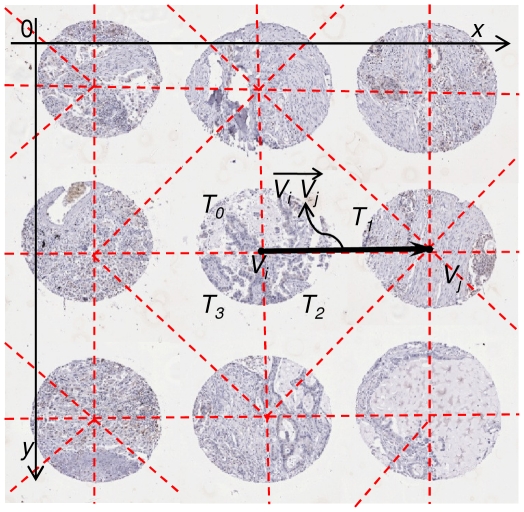
An example showing how to search for the immediate neighbouring TMA core in clockwise and horizontal direction using Delaunay Triangulation.

For an example, to locate 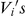 clockwise horizontal neighbours 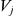 with 2D coordinates 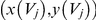, we first locate the set of triangles 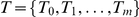 which 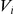 sits as a vertex (example shown in Figure 2, 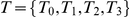). Secondly, we search all edges that use 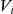 as one of the vertices from each triangle in 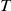 and find the only 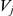 that satisfy the following condition:
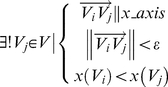
(10)where 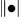 is the magnitude and 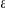 is a global threshold value typically slightly larger than the average centroids distance between two immediate TMA cores (e.g. 1.5 times of the average centroids distance). Then we say that 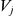 is the direct clockwise horizontal neighbour of 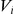.

If we are able to locate the centroids of the left most TMA core in each row of a TMA slide, we can then use the proposed travelling algorithm to identify the logical coordinates of all TMA cores in a horizontal direction. If each identified row of TMA cores can be expressed as 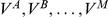, a simple sorting algorithm can then be used to identify their vertical relationship by sorting the arithmetic mean of the y-coordinates 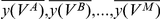. In such a way, the gridding of TMA cores can be achieved.

#### Dealing with Imperfect Data

TMA slides are constructed manually or mechanically. Misalignment of TMA cores often occurs which causes the rotation and/or stretching of the grid. Additionally, some cores are lost and/or are fragmented from individual sections. Furthermore some TMA core segmentation errors are carried over to this stage, e.g. over-segmentation.

**Figure 3:**
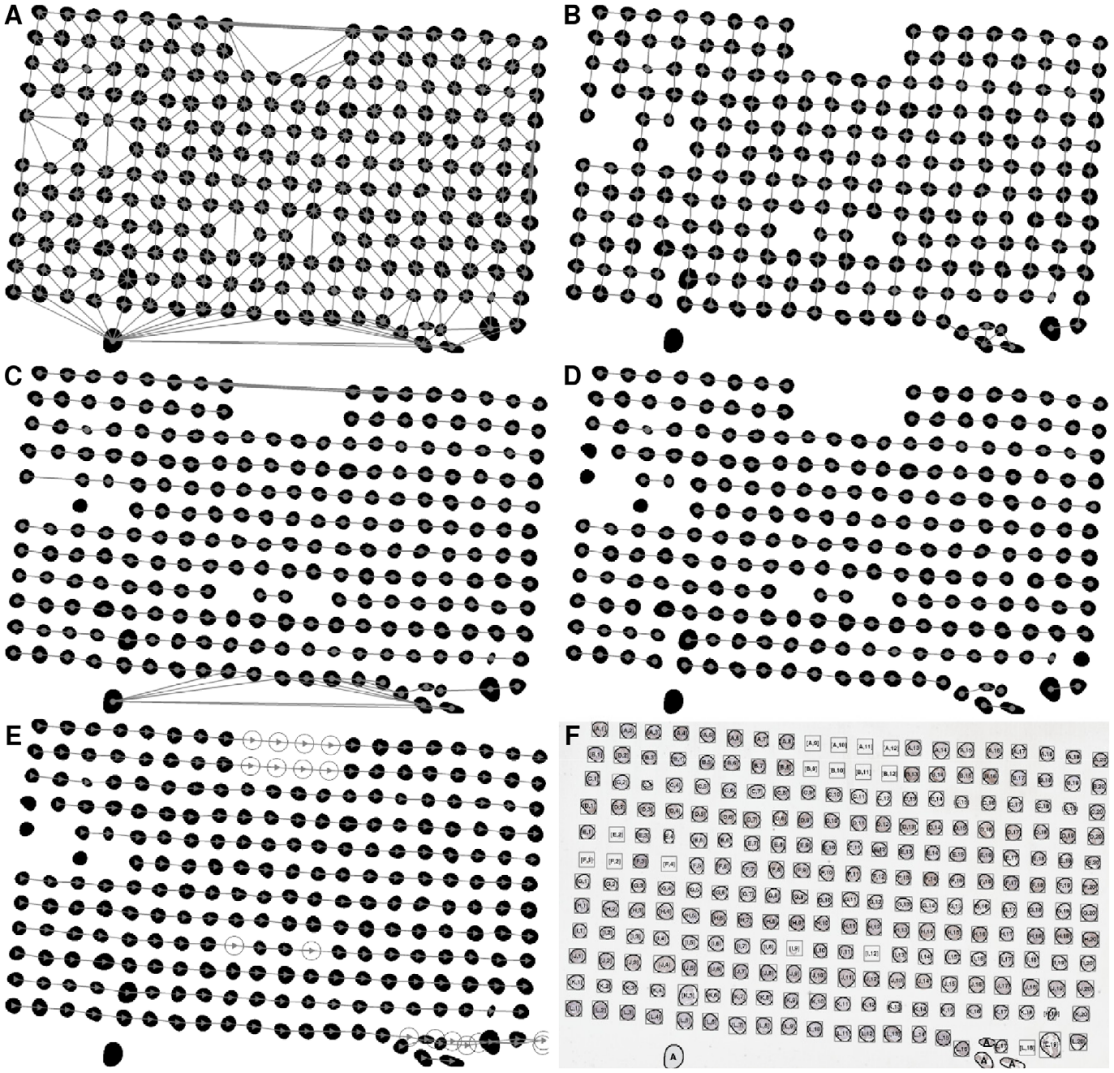
An illustration of TMA core gridding procedure using **Figure 1A** as an example. (A) Result of Delaunay triangulation, (B) Centroids connected via gray lines showing the result of edge length filtering, (C) Centroids connected via gray lines showing the result of edge angle filtering, (D) The candidate centroids for the travelling algorithm, (E) The result of travelling algorithm, (F) To overlap grid index on top of the slide thumbnail, artefacts are marked with an “A”.

An example of such a Delaunay triangulation is shown in Figure 3A. The entire grid is slightly rotated clockwise with a number of missing cores. A few TMA core fragments are presented at the lower right corner. The bottom row of the grid is also skewed. Additionally, an example of an artefact is shown at the bottom left of the image.

#### Edge Length Filtering

As a large amount of the cores and the general grid shape are preserved, most of the triangulations in 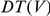 are close to right angled isosceles triangles, with the exception of a small amount of large triangles (caused by missing cores and off-the-grid cores) and thin triangles (mostly at the boundary of the whole grid).

**Figure 4:**
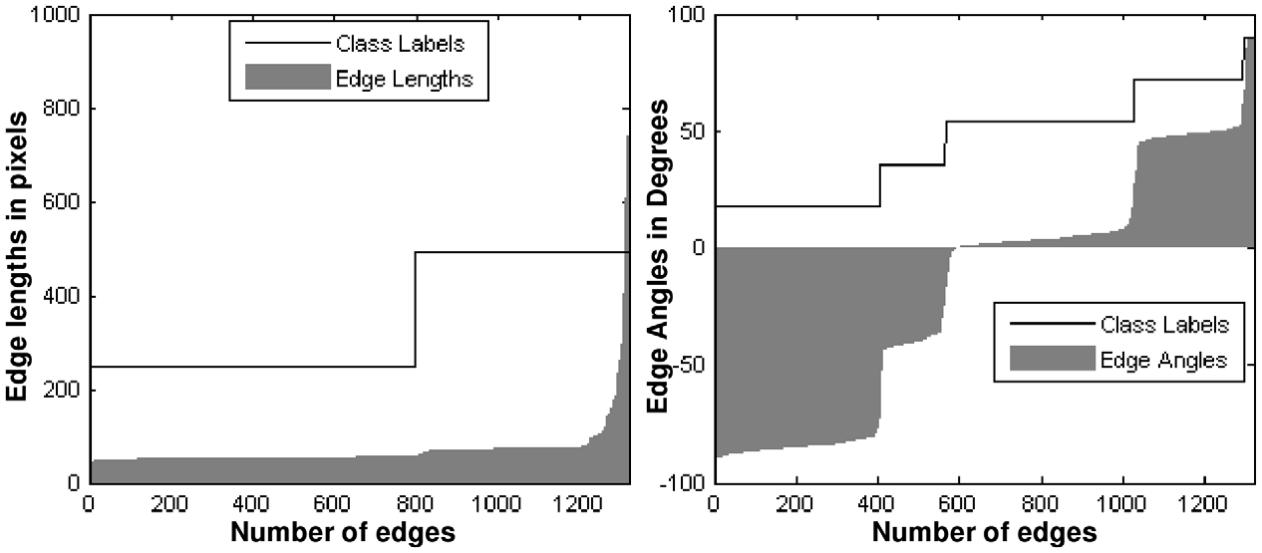
Example of edge length and edge angle filtering. (A) Example of edge length filtering, (B) Example of edge angle filtering.

Consider a right isosceles triangle, the length of both of the two sides are easily identifiable to be as shorter than the length of hypotenuse. Given there are approximately twice as many of sides than the hypotenuses in 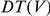, we could sort all the edges 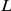 from 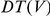 and the smallest 2/3 are largely the near horizontal and vertical edges (denoted as 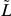). The plot of all edge lengths from 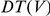 for the example in Figure 3A) is shown in Figure 4A.

To consider the boundary between 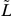 and the remaining edges, the upper outliers of 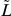 are defined as:
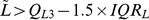
(11)

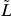
 is further removed to reduce noise. 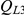 is the third quartile of all edge lengths. An example of the remaining short edges is shown in Figure 3B.

#### Edge Angle Filtering

Rotation changes edge angles for 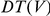. By assuming that the whole TMA core grid is a rigid object and all the angles in 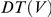 are rotated by 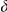 degree clockwise, the majority of angles in 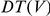 are a subset of:
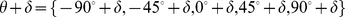
(12)To use a k-mean unsupervised clustering algorithm with 5 clusters and the seeds location to be 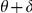, we are able to identify the near-horizontal angle cluster of 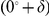 as shown in Figure 3C. The plot of all edge angles from 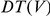 for the example in Figure 3A) is shown in Figure 4B.

#### Traveling

To combine all the edges the results from edge length and edge angle filtering using intersection, we generate an initial template (Figure 3D) for the travelling algorithm. This template contains a set of 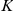 vectors:
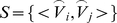
(13)where 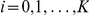, 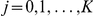, 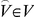, 
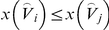
, and every vector in 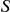 is unique.

The travelling algorithm starts with a vector 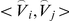 in 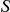 with 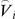 satisfies 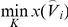. Record 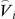 as the first and 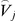 as the second element for a vertex list 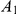. 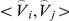 is then removed from 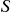.

Afterwards, a loop starts to judge if the terminal point 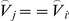 using an exhaustive search, where 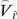 is the initial point for another vector 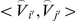 in 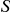. If a match is found, 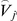 is appended to the end of 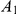, and 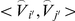 is removed from 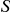. 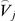 is then re-initialised with the value of 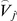. The travelling stops when
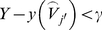
(14)where 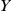 is the width of the image, and 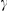 is a small number, typically less than the average core-to-core distance (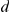). In our experiment, we choose the value of 0.75 times the average core-to-core distance.

When there does not exist such a 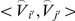, which the current end terminal point 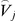 from 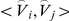 to match up with 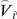 and condition (14) is not satisfied, a circular sector region 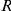 is searched for the existence of such a vertex 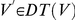. 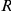 has the radius 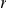 and central angle 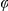, where
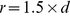
(15)

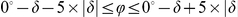
If such a 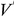 exists, 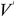 is then appended to the end of 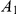. In case of the existence of multiple 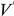, the one which minimise 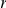 is selected. If 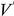 does not exist, an imaginary 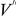 is added to 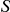, where
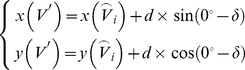
(16)Vector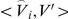 is added to 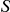 and the travelling continues.

When 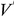 exists in 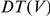 but not in 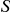, a new circular sector region search will be performed to keep on with the travelling algorithm.

When condition (14) is satisfied, a new start point vector 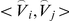 in 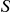 with 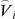 satisfies 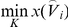 is selected and vertices are stored in a new list 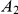. The algorithm continues until 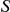 is empty. We then get a set of lists 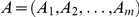. An example is shown in Figure 3E.

#### Gridding

Each list in 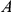 potentially represents a row in a grid. Their row index is obtained by sorted 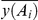 in ascending order. The length of 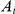 can be a variable. The column index for each vertex in 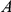 is obtained using the following pattern matching approach (due to the monochrome nature of the y-coordinates in any 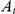, a cross correlation pattern matching approach cannot be used).

For all vertices in a given 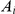, their y-coordinates are approximately equally spaced with distance 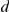. To choose the longest list 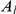. All other lists are then correlated with 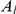 using:
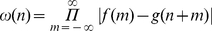
(17)where 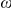 is the proposed correlation vector, 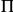 is a median operator. 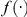 is the function of y-coordinates of 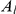, and 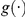 is the y-coordinates of a given 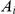.

The y-coordinate offset of 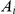 relative to 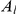 is defined as:
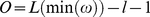
(18)In such a way, all vertices in 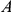 are assigned with a row and column index in a grid 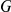.

#### Post-processing

For any of the two neighbouring rows in 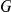, if their row-to-row distance 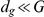, these two rows will be merged and sorted in 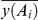 ascending order. When merged, regions exhibiting overlapping and crowding cores are removed and marked as artefacts.

All empty grid elements will be filled with imaginary centroids and their 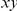-coordinates are obtained through linear interpolation. For all the TMA cores which 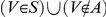, their grid index will be given as the grid index for the imaginary cores which they have the shortest Euclidian distance with.

Finally, boundaries of 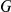 are re-examined and if a whole row/column contains only imaginary cores, the whole row/column is then removed, thereby completing the gridding process. An example of gridding results superimposed on top of segmentation result is shown in Figure 3F.

### Mapping

**Figure 5:**
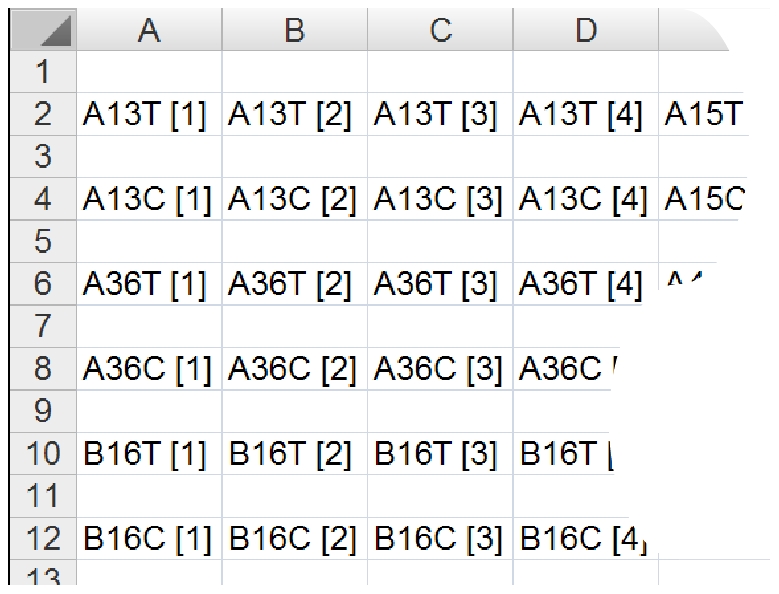
An example of a part of a TMAMap showing TMA core names.

For the biomarker discovery activities with our centre, researchers use a Microsoft Excel file, namely TMAMap, to record TMA core names and associated clinical metadata. These data provide the template for the generation of the physical TMA and therefore provide precise record of the TMA grid pattern as it should appear on the slide. An example is shown in Figure 5. Mapping refers to the task of correlating TMA gridding results with a TMAMap, thereby assigning TMA core names from a TMAMap to each core image.

The first step of mapping is to recognise the orientation of a TMA virtual slide. Once a TMA is sectioned and transferred to a water bath, there are 8 possible orientations [28]. The tissue section can be flipped from left to the right, from the top to the bottom, and/or rotated 90. As the layout of tissue cores can be symmetrical, such as the example showed in Figure 1A, without high-level textual/morphological information, it is very difficult to recognise the orientation of TMA slides. Therefore, in this study the recognition of TMA grid orientation is performed manually.

**Figure 6:**
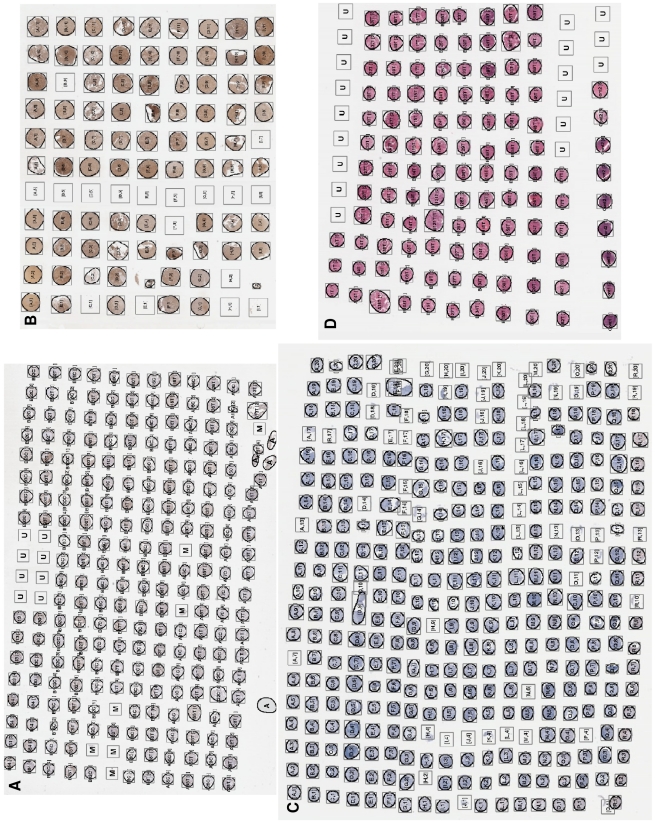
Examples TMA de-arraying results. (A) TMA de-arraying result for the slide with ID 4 from Table 1, (B) Result for the slide with ID 18, (C) Result for the slide with ID 19, (D) Result for the slide with ID 14. *Figure A and D superimposed TMA core names on top of the thumbnails whereas Figure B & C superimposed grid indices on top of the thumbnails.

An Excel-parser is developed to retrieve corresponding TMA names from TMAMap. Each name is correlated with information obtained from TMA core segmentation and gridding, which includes grid index (in both row and column directions), 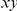-coordinates of TMA cores' top-left corner on the original virtual slide (their 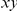-coordinates on the thumbnail ×100), width and height of the TMA core on the original slide. All these correlation information is then recorded into a database [4] or an Excel file for further use. An example demonstrating core name superimposed on top of a TMA thumbnail image is shown in Figure 6A & D, where unoccupied core locations are marked with a “U”, and a missing core is marked with an “M”.

To facilitate further processing of each TMA core, we are able to use the high performance TMA computing platform to rapidly partition the original TMA virtual slide into individual TMA core images at 40× magnification in ≤1 minute [4].

### Additional Pseudo Data for Evaluation

**Figure 7:**
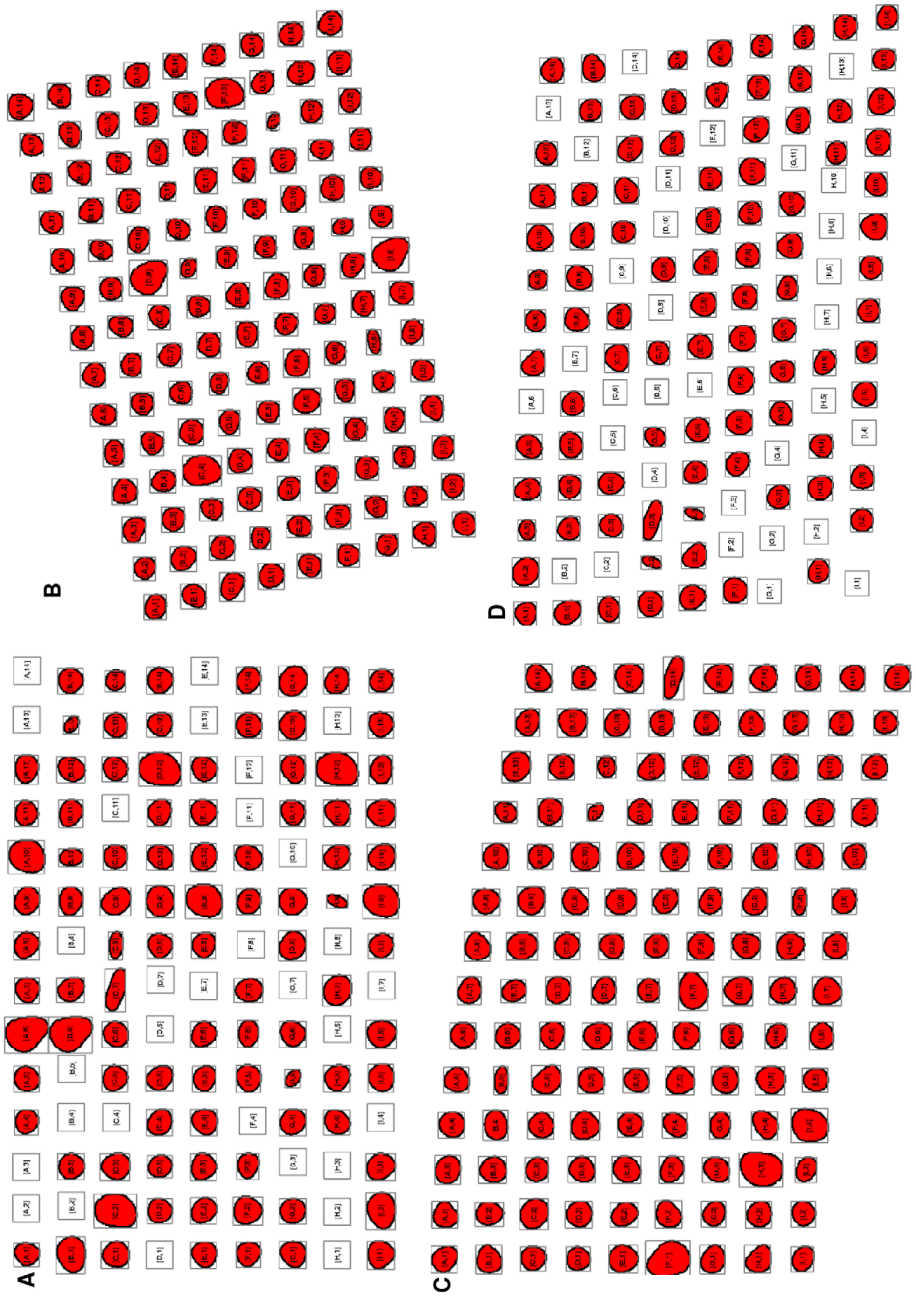
Example TMA de-arraying results using pseudo TMA slide. (A) Result for the pseudo slide from 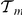 with 31 missing cores, (B) Result for the pseudo slide from 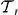 which rotated 15 degree anticlockwise, (C) Result for the pseudo slide from 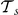 with vertical stretching 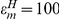, (D) Result for the pseudo slide from 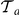 with 32 missing core and no rotation, 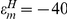 and 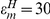. *All figures superimposed grid indices on top of the thumbnails.

To increase the volume of TMA slides for testing and explore a variety of irregularities in TMA slide layouts and their impact on TMA de-arraying algorithms, we artificially created five sets of pseudo TMA slides which contain altogether 113 slides with 13,536 cores in total. Examples of the pseudo TMA slides are shown in Figure 7.

**Figure 8:**
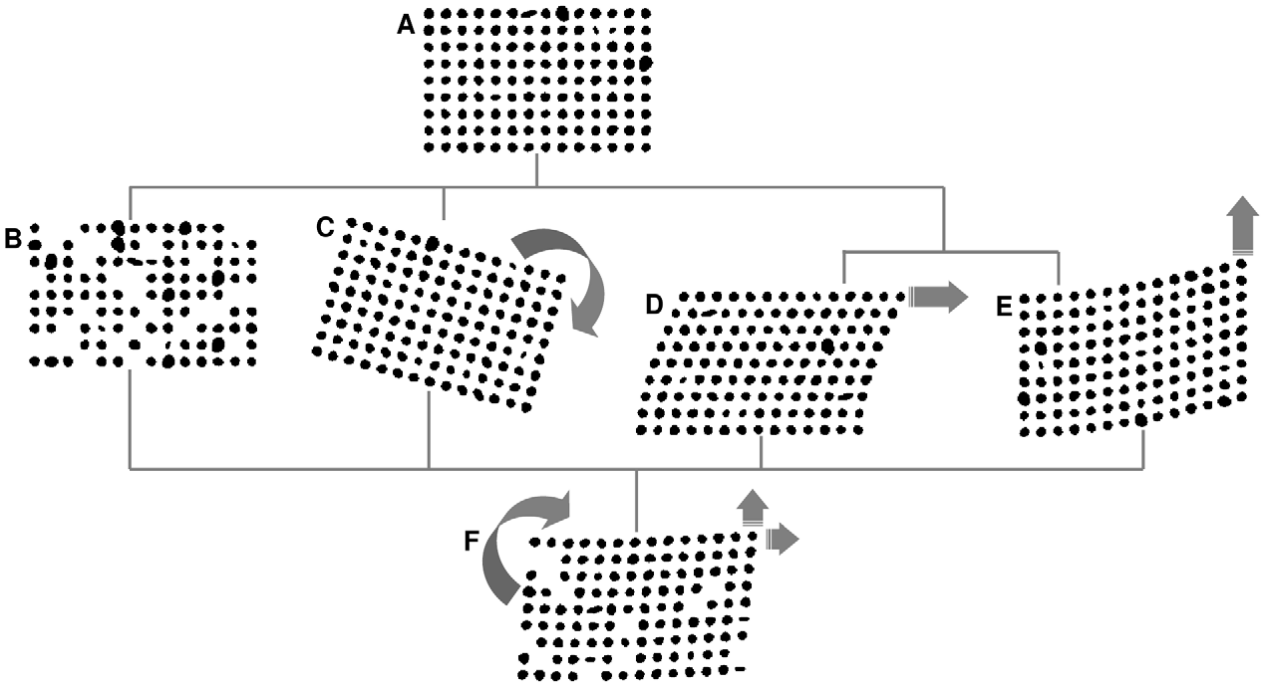
Examples of pseudo test cases. (A) A pseudo test case of a TMA slide with 9 rows and 14 columns of cores (total 126 cores), (B) A pseudo test case reflecting 31 missing cores, (C) A pseudo test case rotated 15 degree clockwise, (D) A pseudo test case stretched at the top right corner with the parameter 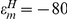, (E) A pseudo test case stretched at the top right corner with the parameter 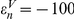, (F) A random test case where the slide has 12 missing cores, and it is rotated 3 degrees anti-clockwise, stretched at the top right corner with the parameters 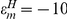 and 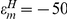.

During the production of TMA slides especially at sectioning and when moving the thin section of paraffin fixation from a TMA recipient block, the regular layout of TMA cores can be altered especially in the following ways: i) TMA cores can be missing, ii) the whole TMA grid can be rotated at varying angles when positioning on a glass slide, iii) the thin TMA tissue section can be stretched at a random direction. These three artefact irregularities are illustrated in Figure 8B–E.

Using the existing TMA tissue samples, we selected a pool of 229 candidate TMA cores. These cores have the diameter of 600 µm and imaged at 40× magnification, however the shape of some of these cores are not completely circular which reflect the reality. These cores are then down-sampled by a factor of 100 in both 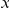 and 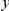 direction, which give the average diameter of 30 pixels per core. These small core images are then converted to be binary images using the proposed TMA core segmentation method described in the Methods section C.1. These candidate cores are then randomly selected and placed on a predefined equally-spaced 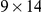 grid with core centroid distance of 50 pixels in both 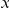 and 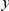 directions to form a regularly spaced TMA slide thumbnail. This thumbnail image contains 126 cores with the image size of 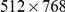 pixels. We name this template TMA slide 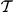 from which irregular TMA layouts could be generated. We artificially created the following five sets of irregular cases, which are the missing core case set 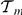 (26 cases), rotation case set 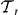 (31 cases), horizontal stretching case set 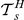 (13 cases), vertical stretching cases 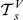 (16 cases) and a mixture set 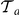 of 27 cases which covers all these 4 degrees of freedom.

### Missing Cores

By randomly removing up to 25% of TMA cores at random locations from 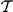, we generated a series of 26 missing-core cases 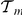 with the number of missing cores ranging from 0 to 31. It is unlikely that this value would be exceeded in real cases. An example is shown in Figure 8B.

### Rotation

By rotating the whole grid layout clockwise and anticlockwise, we obtained a set of 30 rotated TMA cases 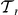 with the angle of rotation within a reasonable range of 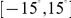, which is 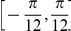. An example is shown in Figure 8C.

### Stretching

To simulate the physical process of TMA stretching, we generated the following model.

If a thin section is stretched horizontally at the bottom right corner of the slide, for each column of TMA cores, the 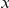-coordinates 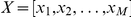 for each of the TMA core in that column would increase, where 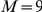 in our case. We use 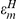 (m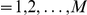) to describe the amount of increments for the 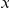-coordinates of each core. As the force is applied on the bottom right corner of the non-rigid section body, 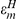 is therefore could be modelled as a non-linear function 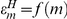. In this study, there was no intention to study in detail the physics of thin section deformation. Therefore we use the following simple differences of cosine signal in the range of 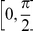 to represent this non-linear deformation:
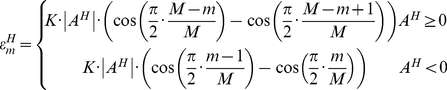
(19)where 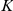 is a scaling factor with the value of 10, and 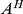 is a user defined horizontal stretching amplitude value. When 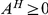, the slide is stretched horizontally at the top right corner, whereas when 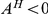, the slide is stretched horizontally at the bottom right corner. Therefore after stretching, the 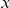-coordinates for a given column are transformed to be 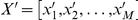, where 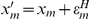.

Similarly, we could also simulate the deformation in the 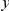 direction by defining the amount of increment 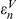 for the 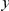-coordinates of a row of TMA cores. 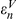 is defined as:
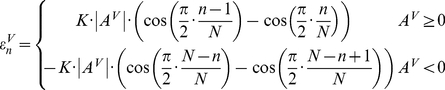
(20)where n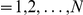, 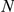 is the number of y-coordinates for a given row of TMA cores, and 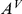 is a user defined vertical stretching amplitude. When 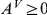, the slide is stretched downwards at the top right corner, whereas when 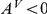, the slide is stretched upwards at the bottom right corner.

By giving different values to 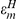, we obtained a collection of 13 horizontally stretched cases 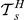, where 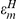 is in the range of 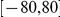. Similarly, a collection of 16 cases 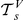 were also generated for vertically stretched cases with 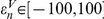. Two examples horizontally and vertically stretched TMAs are shown in Figure 8D & E.

### Random Pseudo Cases

By considering all the above mentioned irregularities, a random set of 27 mixed cases 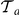 were created. An example is shown in Figure 8F.

## Results

Tests were first performed using 19 TMA tissue virtual slides containing a total of 3129 valid cores. Scanned TMA virtual slides were firstly down-sampled to thumbnail images to be 0.01% of the original slide size (approx. 1,000×1,000 pixels). These thumbnails were then de-arrayed using the proposed three steps approach, segmentation, gridding and mapping. Afterwards, the resulting TMA core information was used to partition the entire original TMA virtual slides into individual TMA core images and archived for further processing. The gridding procedure was also tested using the 113 pseudo slides with 13,536 cores in total.

For the 19 TMA tissue virtual slides, the performance of the proposed de-arraying method was evaluated separately for segmentation, gridding and mapping. Manual evaluation of each step demonstrated the segmentation accuracy of 99.84%, gridding accuracy of 99.81% and mapping accuracy of 99.93%. The robustness of gridding was also evaluated using the 113 pseudo cases, which give the gridding accuracy of 100%. Some examples are shown in Figure 6 & 7.

Taken together these results suggest that the proposed de-arraying method is robust and reliable, and providing an essential tool for the automation of TMA analysis.

### Segmentation

**Figure 9:**
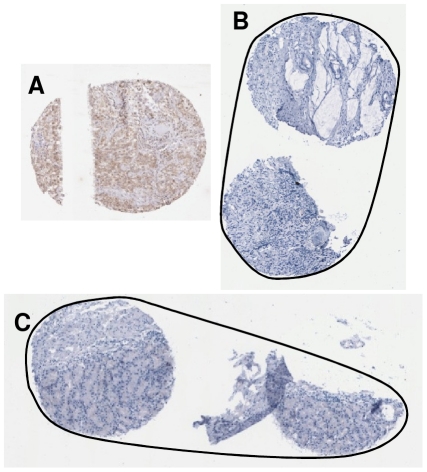
Wrongly segmented TMA cores. (A) A disconnect TMA core, (B) Two neighbouring TMA cores been wrongly segmented as one core, (C) Another example of two neighbouring TMA cores been wrongly segmented as one core. *The contours in Figure B & C are the wrongly recognised boundaries of TMA cores.

**Figure 10:**
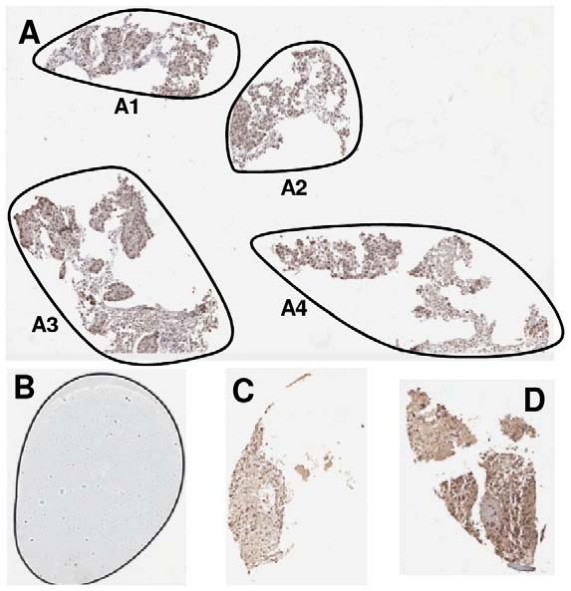
Artefacts which are wrongly recognised as TMA cores during segmentation. (A) Two tissue cores are fragmented into many parts. (B) An air bubble, (C) A fragment of TMA core is wrongly recognised as a TMA core, (D) Another fragment of TMA core is wrongly recognised as a TMA core. *The contours in Figure A illustrate the wrongly segmented TMA core boundaries.

The performance of TMA core segmentation was examined by visually analysing all of the partitioned high resolution TMA core images. A TMA core is considered to be segmented correctly if all parts of the core are segmented and it does not contain tissue/fragments from other cores. All artefacts should not be included and classified as cores either. For the segmentation of TMA cores from thumbnails, given the total of 3129 cores across the 19 TMA slides, only 5 TMA cores were wrongly segmented (Figure 9), and the rest 99.84% cores are correctly segmented as TMA cores. In the analysis, a total of 7 artefacts also presented in the TMA core segmentation results (Figure 10).

For the TMA core shown in Figure 9A, only the tissue fragment on the right is recognised and segmented as a TMA core whereas the fragment on the left is treated as an artefact and removed from segmentation results. Due to geographical closeness and the fragmented nature of the two images in Figure 9B and C, the two TMA cores in each of the image are wrongly recognised as one TMA core.

For the TMA core shown in Figure 10A, two TMA cores were significantly fragmented into a number of tissue parts. They are not suitable to be used in any further TMA experiments and should be discarded. The proposed segmentation algorithm wrongly recognised them as 4 disjoint TMA cores (A1–A4) due to large nature of them. Figure 10B is an air bubble, generated during cover-slipping of the slide, is similar to the size of a TMA core. It was segmented out however further removed during the gridding procedure. Additionally, another two small pieces of tissue (Figure 10C and D) which have been recognised as TMA cores, though these should more appropriately be classified as tissue fragments due to their small nature.

### Gridding

The success of gridding was tested by examining if an identified TMA core was allocated its correct grid index. In our study, we use English alphabetical characters as a row index and Arabic numbers as a column index. As an example, TMA core [B, 8] indicates the TMA core which lies in the 2nd row and 8th column of TMA grid.

An examination across all 19 TMA slides, the majority of TMA cores are gridded correctly with only 6 cores being assigned the wrong grid indices. Thus, the total of 99.81% TMA cores was assigned correctly. These mis-gridded TMA cores are shown in Figure 9B, C and Figure 10A. As can be seen clearly, these mis-gridded TMA cores are the direct results of mis-segmentation from the previous step. As the segmentation method wrongly recognised Figure 9B & C as one TMA core each, the gridding method assigned the two cores in each image with one grid index. Similarly due to segmentation errors, one piece of the tissue fragment (A2 from Figure 10A) is gridded as a TMA core however the rest of tissue fragments (A1, A3 and A4) are successfully recognised and marked as artefacts.

The test on the 113 pseudo test cases suggested that all of the 13,536 pseudo TMA cores were gridded correctly, which gives the gridding accuracy of 100%. The proposed de-arraying method is able to recognise where a single or a continuous number of cores are missing (Figure 7A). For rotated TMA grids (as shown in Figure 7B), our method is also able to de-array such slides correctly without mistaking the logical core coordinates. Stretching creates curves in either horizontal and/or vertical directions hence subsequently alters the straightness of rows and columns where TMA cores sit, our Delaunay triangulation based de-arraying method is proven to be robust in such situations (Figure 7C). Finally, test results also suggest our de-arraying method to be robust and resilient to a random mixture of irregularities, even for the severely altered case shown in Figure 7D.

### Mapping

Comparing with segmentation and gridding, mapping is a straightforward task. It is tested by manual examining if a given TMA core name from the TMAMaps has been assigned correctly to its corresponding TMA core image.

Mapping was tested using the 17 of the total 19 virtual slides (number 1–17) which have a corresponding TMAMap. A total of 2746 cores were tested. Results suggest only 1 mapping error, which is the tissue fragment A2 shown in Figure 10A was given a wrong core name “A13T[4]”. The rest 2745 (99.96%) of TMA cores were all mapped correctly.

## Discussion

In this study, we presented a novel TMA de-arraying technique based on Delaunay triangulation, a computational geometry method. Given the large amount of irregularities within TMA slides, such as missing cores, rotation and stretching, the method from this study is robust and much more resilient than other reported methods such as Hough transform and template matching. Evaluation from this study suggests the proposed three step method results in the accuracy of 99.84% for segmentation, 99.81% for gridding and 99.93% for mapping using real TMA tissue slides.

The gridding of TMA cores were also evaluated using 113 pseudo TMA slides containing 13,536 cores with 100% accuracy. As far as the authors are aware of, this paper represents the first study which defined and evaluated the TMA layout irregularities and their impact on the de-arraying algorithms. The Delaunay triangulation based gridding method presented in this study is able to handle a range of altered TMA layouts, which would cover a large majority of TMA cases in reality. It is capable of dealing with the missing of 24.6% cores, the rotation of grid within the range of 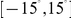, and the stretching in both horizontal (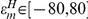) and vertical directions (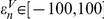).

It is difficult to compare the performance of our study with others simply because there are relatively few de-arraying algorithms in the literature. Most studies reported the overall de-arraying performance [5], [8], rather than the robustness of individual segmentation, gridding and mapping steps. To the best of our knowledge, there are no other studies reporting the performance of TMA core segmentation algorithms. For TMA gridding, Study [5] reported an accuracy of 96.84% using 5878 cores where mis-gridding tends to happen when there is not a complete a row of TMA cores. Study [8] reported 99.59% using 8864 cores with most of mis-gridding to be not-assigned-cores. Using the proposed Delaunay triangulation method, this study achieved 99.81% in gridding using 2747 TMA tissue cores, and 100% in gridding using 13,536 pseudo cores. The proposed gridding method does not depend on complete rows of TMA cores, and as Delaunay triangulation uses the centroid of each TMA core as a vertex, it is highly unlikely that cores are unassigned.

**Figure 11:**
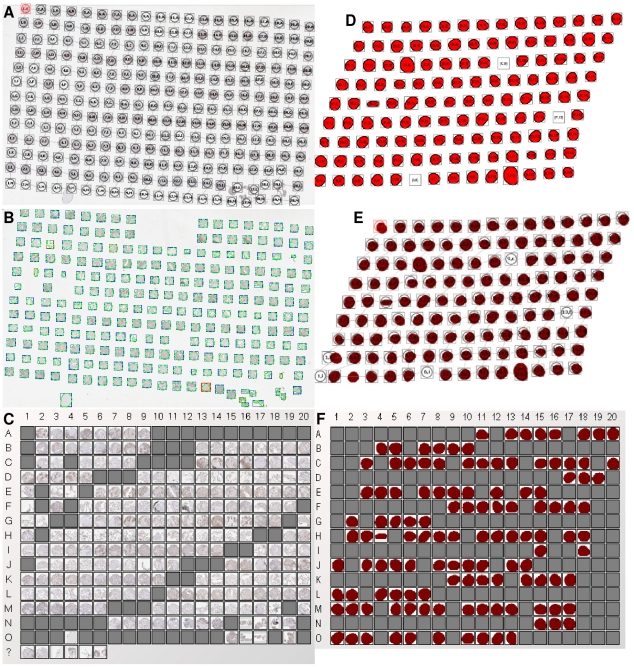
Examples of TMA de-arraying results using Aperio's Spectrum and Definiens' Tissue Studio software. (A) Aperio's de-arraying result for the slide with ID 4 from Table 1, the bottom row ‘M’ represents 20 wrongly recognised TMA cores which are actually background and artefacts, (B) Definiens' segmentation result for the slide with ID 4, (C) Definiens' gridding result for the slide with ID 4, majority of TMA cores (located at in the centre and to the right of the slide) are assigned wrongly as it is unable to recognise the grid rotation from this slide, (D) De-arraying result using our method with a pseudo TMA slide case from 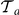 (3 missing cores, no rotation, 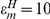 and 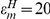), (E) Aperio's Spectrum's de-arraying result on the same pseudo case as Figure D (only 3 cores on the bottom left of the slide are wrongly recognised), (F) Definiens' Tissue Studio's de-arraying result on the same pseudo case as Figure D (A significant amount of segmented cores need to be manually assigned).

We also compared the performance of our de-arraying algorithm with commercially available software, Aperio's Spectrum and Definiens' TissueStudio packages. While the technical details of how these software packages carry out de-arraying are unknown, we were able to compare the overall de-arrayed results directly. Using the same 19 virtual slides with 3129 TMA cores for testing, Aperio's Spectrum also produced very good de-arraying results. 99.68% of the cores are assigned correctly with only 10 wrongly gridded TMA cores, and another 40 background/tissue fragments been wrongly recognised as TMA cores (Figure 11A). For the testing of the 113 pseudo TMA slides with 13,536 pseudo cores, it achieved 99.98% accuracy with only 3 cores were wrongly assigned for a severely stretched case (Figure 11E). However to Aperio's Spectrum software, users need to manually fine tune the location and size of TMA core boundaries (complete circles). In comparison, our de-arraying method contains a robust TMA core segmentation procedure and the exact core boundaries are identified, making it unnecessary to perform the manual core location and boundary adjustments using our method. Definiens' TissueStudio produces excellent TMA core segmentation results by tracking the TMA core boundaries (Figure 11B), however it requires a significant amount user interaction to manually i) identify tissue fragments and artefacts (e.g. the bottom row in Figure 11C marked with a “?”), ii) to correct core locations especially for TMA slides with altered grid layout, such as a rotated and/or stretched grid (Figure 11C & F). In comparison, our de-array method is able to recognise and filter out a significant amount of tissue fragments and artefacts via a robust segmentation approach. By using Delaunay triangulation with the built-in logic to recognise and search for neighbouring cores especially for altered grid layout, the majority of the irregular TMA grid structures are recognised and cores assigned correctly. Additionally, given that our proposed method is able to map each TMA core with its name and associated clinical data through an association with the underlying TMAMap, this represents an enormous benefit over existing commercial systems.

In our experience, given the enormous variability that is encountered in TMA construction, it is difficult to achieve 100% accuracy of TMA core de-arraying assignment. For this reason, visual inspection to ensure appropriate gridding results remains important. However, with more robust algorithms, such as the one presented in this study, the amount of manual correction will be significantly reduced.

The development of artificially produced pseudo TMA slides showing a wide range of artefacts is important. This allows rapid prototyping and evaluation of new algorithms for TMA gridding and these are now made available publically for others to access.

For future work, the robustness of the described TMA de-arraying method can be further improved by using the existing grid information that exists in the pre-defined TMAMap. Here, the layout, number of rows and columns etc are all pre-defined and could be used to check the validity of the de-arraying results. Additionally, the edge angle filtering method, which was described in the Methods section C.2.d, uses a simple k-means clustering approaching with 5 clusters. In extreme situations the number of edge angle clusters could be less than 5 (in a artificially created regular pseudo slide, e.g. the edge angle of 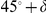 is missing), a number of cluster number determination methods could be applied, such as the information theoretic approach [29], and a genetic algorithm which optimises the silhouettes [30].

In Summary, in this study we have developed a TMA de-arraying method which recognises TMA cores and assigns them to their corresponding grid index (and core names when available). The proposed method is logically sound and results on real TMA images and pseudo datasets indicate that it is robust. The correct assignment of TMA cores is a first important step for the automated and high throughput analysis of TMAs using computer based image analysis. This is now becoming the cornerstone of target validation, drug evaluation and biomarker discovery in animal and human tissues.

### Availability

For comparison studies and evaluations, all the pseudo TMA data are freely available at https://picasaweb.google.com/117531880452844036890/TMADeArrayingPseudoData. The result mark-up images using our proposed TMA de-arraying method can be found at https://picasaweb.google.com/117531880452844036890/TMADeArrayingPseudoDataResults.
